# Proteomics As a Tool for Studying Bacterial Virulence and Antimicrobial Resistance

**DOI:** 10.3389/fmicb.2016.00410

**Published:** 2016-03-31

**Authors:** Francisco J. Pérez-Llarena, Germán Bou

**Affiliations:** Servicio de Microbiología-INIBIC, Complejo Hospitalario Universitario A CoruñaA Coruña, Spain

**Keywords:** resistance, diagnostic, antibiotic, virulence, proteomics, bacteria

## Abstract

Proteomic studies have improved our understanding of the microbial world. The most recent advances in this field have helped us to explore aspects beyond genomics. For example, by studying proteins and their regulation, researchers now understand how some pathogenic bacteria have adapted to the lethal actions of antibiotics. Proteomics has also advanced our knowledge of mechanisms of bacterial virulence and some important aspects of how bacteria interact with human cells and, thus, of the pathogenesis of infectious diseases. This review article addresses these issues in some of the most important human pathogens. It also reports some applications of Matrix-Assisted Laser Desorption/Ionization-Time-Of-Flight (MALDI-TOF) mass spectrometry that may be important for the diagnosis of bacterial resistance in clinical laboratories in the future. The reported advances will enable new diagnostic and therapeutic strategies to be developed in the fight against some of the most lethal bacteria affecting humans.

## Introduction

Bacterial diseases continue to be a major cause of death throughout the world as a result of the emergence of new infectious agents, increased transmission due to human migration, and the development of antibiotic resistance (Beceiro et al., 2013). Novel antibiotics and therapies are urgently needed to control these infections, together with new rapid and reliable diagnostic techniques for characterizing resistant strains. Matrix-Assisted Laser Desorption/Ionization-Time-Of-Flight (MALDI-TOF) mass spectrometry techniques may be useful for this task. The present review considers some recent advances in the diagnosis of microbial resistance. Examination of protein profiles by proteomic analysis has become an essential tool for studying the basic mechanisms of bacterial resistance and virulence. This has led to a better understanding of the biology of pathogens that cannot be investigated by reductionist or even genomic studies (e.g., global post-translational protein modifications, subcellular protein location, and protein turnover rates, which are also considered in this review; Chao and Hansmeier, 2012).

Proteomic techniques are continuously being developed and there is now a great diversity of methods and applications available (Van Oudenhove and Devreese, 2013; Otto et al., 2014). The improved sensitivity of mass spectrometers, together with upgraded sample preparation and protein fractionation technologies, has enabled a more complete study of proteomes. In the past, most quantitative proteomic studies were performed by 2D-PAGE, particularly after the incorporation of immobilized pH gradients and of the difference fluorescent labeling method (DIGE). The gel free method then became more popular and metabolic labeling was included in quantitative analysis. In this labeling method, cells are grown under appropriate conditions in media supplied with either a light or heavy stable isotope of a nutrient, commonly a nitrogen source or an amino acid. The so-called stable isotope labeling by amino acids in cell culture (SILAC) strategy now prevails (Ong et al., 2002). As a substitute to metabolic labeling, differential analysis can be carried out by chemical labeling. Isotope-coded affinity tags (ICAT) covalently label cysteines in extracted proteins with either a light or heavy (deuterium containing) conformation of the ICAT indicator (Gygi et al., 1999). Today, the use of multiple isobaric tags is prevailing among chemical isotope labeling methods. Some products such as isobaric tags for relative and absolute quantification (iTRAQ) and tandem mass tags (TMT) are commercially available (Thompson et al., 2003; Ross et al., 2004). A similar strategy, known as isotope-coded protein label (ICPL), in which both N-termini and lysine side chains are labeled, has been developed for use at the protein level (Schmidt et al., 2005). Recent progress in the development of high throughput and automation of liquid chromatography–mass spectrometry (LC–MS) instruments and in particular progress toward the use of novel algorithms to handle LC–MS data is facilitating the application of quantitative proteomics using label-free strategies. The methods, in which synthetic proteotypic peptides labeled with stable isotopes are used as the ideal internal standard, are becoming particularly popular. Thus, these internal standard peptides are used to measure the total quantity of individual proteins of interest after digestion by selected reaction monitoring (SRM)-MS measurements (Gerber et al., 2003). The various advantages and disadvantages of the different protocols are summarized in Table 1 (Tiwari and Tiwari, 2014).

**Table 1:** **Different quantitative proteomic approaches with the associated advantages and disadvantages**.

**Proteomic tools**	**Advantage**	**Disadvantage**
**GEL-BASED METHODS**		
2DE	(1) Simple (2) Robust (3) Suitable for MS analysis	(1) Involves large amount of sample (2) Low throughput (3) Poor recovery of hydrophobic proteins (4) High inter-gel variability
2-DIGE	(1) Multiplexing (2) Better quantitation (3) Minimal gel to gel variation	(1) Expensive Cy dyes (2) Poor recovery of hydrophobic proteins (3) Difficulty in separating low molecular weight compounds
**GEL-FREE METHODS**		
SILAC	(1) High throughput (2) Robust (3) Sensitive and simple	(1) Only suitable for tissue culture models (2) Costly reagents (3) Not applicable to tissue sample
ICAT	(1) Selectively isolates peptide (2) Compatible with any amount of protein (3) Complexity of the peptide mixture is reduced	(1) Cannot identify proteins with less than eight cysteines (2) Large ICAT label (≈500 Da) (3) Post-translational modification information is frequently lost
iTRAQ	(1) Applicable to versatile samples (2) Multiplexing (3) Better quantitation	(1) Involves high amount of sample (2) Incomplete labeling (3) Expensive reagents
ICPL	(1) High-throughput quantitative proteome profiling on a global scale (2) Able to detect post-translational modifications and protein isoforms (3) Applicable to protein samples such as tissue extracts or body fluids	(1) Isotopic effect of deuterated tags interferes with retention time of the peptides labeled during LC
Label-free	(1) Involves less amount of sample (2) Higher proteome coverage (3) Avoids labeling	(1) High throughput instrumentation (2) Not suitable for low abundant proteins (3) Incomplete digestion may introduce error (4) Multiplexed analysis not possible in one experiment
SRM	(1) Highly sensitive, quantitatively accurate and highly reproducible (2) Protein detection is relatively rapid and straightforward (3) Enables detection of non-abundant (>10 ng/ml) proteins (4) Quantification of post-translational modifications	(1) Limited broad scale application because of difficulty in generating high-quality SRM assay (2) Sensitivity is not comparable to immunological assays (3) Detection and quantification of non-abundant proteins (i.e., ~10 ng/ml or less)

*Obtained from Tiwari and Tiwari (2014) and reprinted with permission from the publisher*.

The potential application of proteomics to bacterial pathogen research is huge. However, this review will only consider some of these applications and will specifically address bacteria (rather than fungi). The basic knowledge concerning resistance and virulence obtained recently through proteomic technology should be useful for developing new diagnostic and therapeutic applications for the treatment of infectious diseases.

## Proteomics for studying interactions between bacteria and antibiotics

### General aspects

Antibiotic resistance has become a serious problem in the past two decades and an understanding of the mechanisms of the process is necessary to extend the life of current antibiotics and to enable discovery of novel targets (Lee C. R. et al., 2015). We will describe how antibiotic resistance in bacteria is studied by proteomic techniques. In proteomics, analysis of the total protein content of cells is usually carried out after sublethal contact between selected antibiotics and a pair of isogenic resistant and susceptible bacterial strains. In some cases, intermediate phenotypes are also studied. Resistant strains may be clinical strains or strains obtained *in vitro* (Park et al., 2016). The proteomic response is usually specific to each antibiotic, but proteins involved in energy and nitrogen metabolism, protein and nucleic acid synthesis, glucan biosynthesis, and stress response are often affected (Park et al., 2016). The findings of proteomic studies are commonly confirmed by genomic and/or transcriptomic analysis of the strains and in some cases also by study of the response of strains in which relevant genes are inactivated by gene replacement technology (Lima et al., 2013).

We will describe essential studies and novel findings as well as some proteomic studies involving the main bacterial antibiotic families.

### Antibiotics targeting the cell wall

#### Beta-lactams

Resistance to beta-lactam antibiotics is one of the types of resistance most commonly studied by proteomics methods (Lima et al., 2013). The beta-lactams antibiotics (e.g., penicillin, cephalosporin, carbapenens, monobactam, and beta-lactamase inhibitors) may disturb the synthesis and/or stability of the cell envelope, thus disrupting cell-wall biogenesis and leading to loss of selective permeability and osmotic integrity, finally causing bacterial cell death (Waxman and Strominger, 1983). The main mechanism of resistance to beta-lactam antibiotics is the presence of antibiotic hydrolyzing proteins, known as beta-lactamases (Pérez-Llarena and Bou, 2009). Other important mechanisms include the imbalance in transport proteins such as efflux pumps and porins and alteration in the penicillin binding protein targets (Poole, 2004). The increased use of antibiotics has generally led to the prevalence of some important resistance strains such as penicillin resistant *Streptococcus pneumoniae*, methicillin resistant *Staphylococcus aureus*, and extended spectrum beta-lactamase (ESBL), and cabapenemase-producing *Enterobacteriaceae, Pseudomonas aeruginosa*, and *Acinetobacter baummanni* (Boucher et al., 2009).

One of the earliest proteomic studies was an investigation of ampicillin resistant *Pseudomonas aeruginosa*, in which novel porins involved in resistance were discovered (Peng et al., 2005). Study of resistance to piperacillin/tazobactam in *Escherichia coli* has revealed reduced expression of porin OmpX and increased expression of TolC (Dos Santos et al., 2010). In the case of the penicillin-tolerant Gram-positive *Streptococcus pyogenes*, overexpression of murein metabolism proteins and general alteration of bacterial physiology were observed (Chaussee et al., 2006).

Alterations in cell physiology and overexpression of catalase and superoxide dismutase were detected in methicillin resistant *S. aureus*. The role of alanine dehydrogenase was indicated as being important in antibiotic resistance (Monteiro et al., 2012). Adaptation to oxacillin in *S. aureus* has recently been investigated (Solis et al., 2014). These authors concluded that proteins involved in capsule formation, peptidoglycan biosynthesis, and wall remodeling are regulated in response to antibiotics. Spectral counting-based label-free quantitative proteomics has been applied to study global responses in methicillin-resistant *Staphylococcus aureus* (MRSA) and methicillin susceptible *S. aureus* treated with subinhibitory doses of oxacillin (Liu et al., 2014). Beta-lactamase and penicillin-binding protein 2a were uniquely upregulated in oxacillin-treated MRSA (Table 2). Analysis of the inner membrane fraction of carbapenem resistant *A. baumanni* has shown an association with beta-lactamase AmpC and OXA-51 production as well as metabolic enzymes, elongation factor Tu, and ribosomal proteins (Tiwari et al., 2012; Tiwari and Tiwari, 2014).

**Table 2:** **Pathway enrichment study by Database for Annotation, Visualization, and Integrated Discovery (DAVID) of the differentially expressed proteins in oxacillin-treated MRSA and MSSA compared with their untreated controls**.

**Pathway**	**Oxacillin treated MRSA Control**	**Oxacillin treated MSSA Control**
Alanine, aspartate, and glutamate metabolism	−	
Beta-lactam resistance	+	
Peptidoglycan biosynthesis	+	+
Pantothenate and CoA biosynthesis	+	+
Aminoacyl-tRNA biosynthesis		−
Pentose and glucuronate interconversions		−
ABC transporters		−
Porphyrin and chlorophyll metabolism		−
Two component system		+
Nitrogen metabolism		+
Oxidative phosphorylation		+
RNA degradation		+
Nicotinate and nicotinamide metabolism		+
Arginine and proline metabolism		+
Ribosome		+
Pyrimidine metabolism		+
Purine metabolism		+
Nucleotide excision repair		+
Pyruvate metabolism		+
Cytrate cycle (TCA cycle)		+

*Obtained from Liu et al. (2014) and reprinted with permission from the publisher. (+) Upregulated; (–) Downregulated*.

#### Glycopeptides

The glycopeptide vancomycin acts by inhibiting peptidoglycan synthesis. It binds to the DAla-DAla terminus of the nascent peptidoglycan, thus blocking its correct synthesis. In *Enterococcus* spp., a substitution of the DAla residue from peptidoglycan termini by D-lactose or D-Serine has been detected as the main mechanism of resistance to vancomycin. In *S. aureus*, a more complex scenario involving different enzymes and gene clusters implicated in vancomycin resistance has been proposed. Some resistant strains such as vancomycin resistant *Staphylococcus aureus* (VRSA) and vancomycin resistant *enterococci* (VRE) are of serious clinical concern (Lima et al., 2013).

In the first proteomic study of vancomycin resistant *Enterococcus faecalis*, Wang et al. (2010) examined a reference strain (V583) and a clinical isolate (V309) in the presence and absence of vancomycin. These authors found that proteins involved in vancomycin resistance functions, virulence factors, stress, metabolism, translation, and conjunction were regulated. Proteomic profiles of vancomycin resistant *E. faecium* SU18 strain treated and not treated with vancomycin have recently been obtained (Ramos et al., 2015). Fourteen proteins were differentially expressed in SU18. Proteins involved in the vancomycin resistance mechanisms were upregulated in the presence of vancomycin, while metabolism-related proteins were downregulated, leading to compensatory effects. Differential expression of proteins has been observed in vancomycin resistant *S. aureus*, thus distinguishing vancomycin intermediate (VISA) type strain Mu50 and vancomycin resistant strains (Drummelsmith et al., 2007). More recently, the proteomic profile of a group of heterogeneous vancomycin-intermediate *Staphylococcus aureus* (hVISA) was compared with that of vancomycin susceptible *S. aureus* (Chen et al., 2013). The study initially detected five upregulated proteins in hVISA, although only one was definitely confirmed by real time quantitative reverse transcription PCR (qRT-PCR): the protein encoded by the isaA gene involved in cell wall biogenesis.

### Antibiotics targeting protein synthesis

#### Chloramphenicol

Chloramphenicol acts by binding to the 50 S ribosome subunit. Three mechanisms of resistance to chloramphenicol are known: reduced membrane permeability, mutation of the 50S ribosomal subunit, and production of chloramphenicol acetyltransferase (Civljak et al., 2014). Li et al. (2007) observed differential expression of 10 membrane proteins, including TolC, OmpC, OmpW, and OmpT, in chloramphenicol resistant *E. coli*. A more recent study demonstrated overexpression of two different efflux pumps in chloramphenicol resistant strains of *Burkholderia thailandensis* obtained in the laboratory (Biot et al., 2011).

#### Linezolid

In the case of linezolid, an oxazolidinone that binds to the 23S rRNA (Ribosomal ribonucleic acid), different resistance mechanisms have emerged, including increased expression of ABC transporters, mutations in 23S rRNA, mutations in ribosomal proteins L3 and L4, and mutations in an RNA methyltransferase (Lee C. R. et al., 2015). Proteomic and transcriptomic screening of linezolid revealed a possible increase in the metabolism and transport of carbohydrates in some linezolid-resistant *S. pneumoniae* mutants (Feng et al., 2011). Several glycolytic proteins were overexpressed in the resistant strains, along with other enzymes and transporters involved in sugar metabolism.

#### Tetracyclines

The tetracycline family of antibiotics inhibits aminoacyl tRNA binding to the mRNA-ribosome complex. Cells become resistant to tetracycline via at least three mechanisms: enzymatic inactivation of tetracycline, efflux, and ribosomal protection (Falagas et al., 2015).

Yun et al. (2008) used 2-DE/MS-MS (Two-dimensional tandem mass spectrometry) and 1-DE/LC/MS-MS (One-dimensional Liquid chromatography tandem mass spectrometry) techniques to study the surface proteome of *A. baumannii* DU202 (which is highly resistant to tetracycline) after treatment with subminimal inhibitory concentrations (subMIC) of tetracycline. These authors observed that OmpA38, CarO, OmpW, and other Omps were increasingly secreted on exposure to tetracycline. This indicates an important role for Omps in overcoming antibiotic-induced stress.

A parallel proteomic approach has been applied in the analysis of sarcosine-insoluble outer membrane fraction of *P. aeruginosa* responding to ampicillin, kanamycin, and tetracycline resistance. The effects on expression levels were variable and expression of OprG decreased as expression of OprF, Oprl, Omp, and MexA increased (Peng et al., 2005). In an interesting study, Lin et al. (2014) labeled the differential proteome of *E. coli* K12 BW25113 in response to chlortetracycline stress with isobaric tags and applied quantitative proteomics technology for relative and absolute quantitation of the labeling. Crucial metabolic pathways such as the tricarboxylic acid cycle, pyruvate metabolism, and glycolysis/gluconeogenesis fluctuated greatly. The ribosome protein complexes contributing to the translation process were generally elevated under conditions of chlortetracycline stress, which is known to be a compensatory mechanism caused by the action of chlortetracycline on the ribosome.

#### Aminoglycosides

The aminoglycoside antibiotic family interrupts protein synthesis by blocking the small 16S subunit of the bacterial ribosome. Three mechanisms of aminoglycoside resistance are known: reduced uptake or decreased cell permeability, alterations at the ribosomal binding sites, and production of aminoglycoside modifying enzymes (Jackson et al., 2013).

In an initial study, a subproteomic approach was used to characterize the outer membrane proteins of streptomycin resistant *E. coli*. TolC, OmpT, and LamB were upregulated and FadL, OmpW and a location-unknown protein Dps were downregulated in the streptomycin-resistant *E. coli* strain (Li et al., 2008). Another study analyzed and compared the protein profile of whole cell extracts from *Mycobacterium tuberculosis* clinical isolates susceptible and resistant to streptomycin. On comparing 2DE patterns, nine proteins were consistently overexpressed in streptomycin resistant isolates and were identified. Moreover, *in silico* docking analysis revealed significant interactions between these proteins and streptomycin (Sharma et al., 2010). In a native/SDS-PAGE based proteomic study, low levels of NarG and NarH, two components of respiratory nitrate reductase (Nar), were observed in streptomycin, gentamicin, ceftazidime, tetracycline, and nalidixic acid-resistant *E. coli* strains (Ma et al., 2013).

Nabu et al. (2014) compared the protein expression profiles of a high-level spectinomycin-resistant (clinical isolate) and a susceptible (reference strain) *Neisseria gonorrhoeae* after treatment with subminimal inhibitory concentrations (subMICs) of spectinomycin. The 50S ribosomal protein L7/L12, an essential component for ribosomal translocation, was over-expressed in both strains, indicating that compensatory mechanisms may work in response to antibiotics that inhibit protein synthesis. Proteomics techniques have been used to establish the effects of gentamicin on the proteomes of aerobic and oxygen-limited *E. coli* (Al-Majdoub et al., 2013). Ribosomal proteins L1, L9, L10, and S2 were upregulated under both conditions, and the authors postulated that these are candidate drug targets for the development of synergistic combinations with gentamicin.

Kanamycin and amikacin resistant isolates of *M. tuberculosis* have been studied by proteomic analysis. Twelve proteins, two of which are of unknown function, were upregulated in both antibiotic-resistant isolates. All of the proteins were cellular proteins. Kanamycin and amikacin interacted correctly with the proteins according to molecular docking studies. Kumar et al. (2013) suggested that two of them were putative iron regulation/metabolism related proteins, thereby indicating the role of iron in conferring resistance to second-line drugs. In a recent proteomic and western blotting study of the *E. coli* K-12 outer membrane (OM) proteins involved in kanamycin resistance, Li H. et al. (2015) observed upregulation of some OM proteins such as Tolc, TsX, and OstA, and downregulation of MipA, OmpA, FadL, and OmpW OM proteins in the kanamycin resistant *E. coli* K-12 strain. These authors concluded that MipA is a novel OM protein implicated in antibiotic resistance.

#### Macrolides

Macrolide antibiotics act by binding reversibly to the P site on the subunit 23 S of the bacterial ribosome. The primary means of bacterial resistance to macrolides is by post-transcriptional methylation of the 23S bacterial ribosomal RNA. Two other rarely observed types of acquired resistance include the production of drug-inactivating enzymes (esterases or kinases) and the production of active ATP (Adenosine triphosphate)-dependent efflux proteins that transport the drug outside of the cell (Cornick and Bentley, 2012).

In an early study, Cash et al. (1999) examined the proteins synthesized by erythromycin-susceptible and erythromycin-resistant *S. pneumoniae*. These authors used peptide mass mapping to identify a 38,500 Dalton protein upregulated in resistant strains as glyceraldehyde-3-phosphate dehydrogenase (GAPDH). Considering this as the possible cause of resistance against erythromycin, the authors proposed an increment in energy production for the efflux system.

In comparative proteomic analysis of isolated sarcosine-insoluble outer membrane protein (OMP) fractions from clarithromycin-susceptible and resistant *Helicobacter pylori* strains, Smiley et al. (2013) showed that iron-regulated membrane protein, UreaseB, EF-Tu, and a putative OMP were downregulated; the HopT (BabB) transmembrane protein, HofC and OMP31 were upregulated in clarithromycin-resistant *H. pylori*, revealing that alteration of the outer membrane protein profile may be a novel mechanism involved in clarithromycin resistance in *H. pylori*.

### Antibiotics targeting DNA or RNA synthesis

#### Metronidazole

Metronidazole is an antibiotic of the nitroimidazole class that inhibits nucleic acid synthesis by disrupting the DNA of microbial cells. Multiple mechanisms or resistance have been described in *Bacteroides fragilis* and *H. pylori* (Chong et al., 2014)

After analysis of the protein profiles of a derivative of *H. pylori* strain 26695, which is resistant to moderate levels of metronidazole, McAtee et al. (2001) proposed that the ability of the mutant strain to increase various isoforms of alkylhydroperoxide reductase during exposure to metronidazole is critically important in producing the resistant phenotype. In a metronidazole-resistant strain derived from *B. fragilis* ATCC 25285, the proteomic changes affected a wide range of metabolic proteins, including lactate dehydrogenase (upregulated) and flavodoxin (downregulated), which may be involved in electron transfer reactions: the enzymatic activity of the pyruvate-ferredoxin oxidoreductase (PorA) complex was also found to be impaired (Diniz et al., 2004).

Alterations in the metabolic pathway involving pyruvate-ferredoxin oxidoreductase were also found in a multidisciplinary analysis of a non-toxigenic *Clostridium difficile* strain with stable resistance to metronidazole (Moura et al., 2014).

Another recent proteomic analysis revealed regulation of DNA repair proteins, putative nitroreductases and the ferric uptake regulator (Fur) in a NAP1 *C. difficile* clinical isolate resistant to metronidazole (Chong et al., 2014).

#### Rifampicin

Rifampicin inhibits bacterial DNA-dependent RNA synthesis by inhibiting bacterial DNA-dependent RNA polymerase. Resistance to rifampicin arises from mutations that alter residues of the rifampicin binding site on RNA polymerase, resulting in decreased affinity for rifampicin. Resistant mutations map to the *rpoB* gene, encoding RNA polymerase beta subunit (Goldstein, 2014).

Neri et al. (2010) found that 23 proteins were differentially expressed in two rifampicin resistant and one susceptible meningococcus. Increased expression of some of the proteins involved in the major metabolic pathways, mainly pyruvate catabolism and the tricarboxylic acid cycle, was observed. Decreased expression of proteins involved in gene regulation and in polypeptide folding was also observed. Sandalakis et al. (2012) analyzed rifampicin resistance in a rifampicin resistant strain of *Brucella abortus* 2308 developed *in vitro*. The resistant strain contained 39 differentially regulated proteins, most of which are involved in various metabolic pathways. The authors suggested that, apart from mutations in the rpoB gene, rifampicin resistance in *Brucella* mainly involves excitation of several metabolic processes and possible use of the already existing secretion mechanisms at a more efficient level.

#### Quinolones

The fluoroquinolones are the most commonly used family of quinolones in clinical settings. First and second generation fluoroquinolones selectively inhibit the topoisomerase II ligase domain. Third and fourth generation fluoroquinolones are more selective for the topoisomerase IV ligase domain, and thus have enhanced Gram-positive coverage. Three mechanisms of resistance to quinolones are known: efflux pumps; proteins that can bind to DNA gyrase, protecting it from the action of quinolones; and mutations at key sites in DNA gyrase or topoisomerase IV, which decrease their binding affinity and thus decrease the effectiveness of the drug (Redgrave et al., 2014). Two early proteomic studies considered the effects of fluoroquinolones on *P. aeruginosa* and *Salmonella enterica*. In the first of these, expression of a probable ATP-binding component of ATP binding cassette (ABC) transporter was observed in ciprofloxacin-intermediate and resistant strains, but not in the sensitive strain (Zhou et al., 2006). The authors of the second study, involving the *S. enterica* multiple antibiotic resistant strain, suggested that the increased expression of AcrAB/TolC was associated with resistance, while increases in e.g., F1F0-ATP synthase and Imp were a response to fluoroquinolone (Coldham et al., 2006). In a proteomic study of nalidixic acid (NA) resistance in *E. coli*, Lin et al. (2008) observed upregulation of TolC, OmpT, OmpC, and OmpW and downregulation of FadL in resistant strains. In a broader search for mechanisms at the protein level that confer resistance to fluoroquinolones, Vranakis et al. (2011) compared the proteomes of fluoroquinolone-susceptible *Coxiella burnetii* and fluoroquinolone-resistant samples of the bacterium (developed *in vitro*). The study revealed differential expression of 15 bacterial proteins involved in different cellular processes, suggesting that the mechanism of antibiotic resistance in the bacterium is a multifaceted process.

### Antibiotics targeting cell membranes

#### Daptomycin

Daptomycin is a lipopeptide that interacts with phosphatidylglycerol in the bacterial membrane, leading to the formation of holes that leak intracellular ions. Until now, specific genetic determinants of the daptomycin-resistant strains remain to be elucidated (Lee C. R. et al., 2015). Fischer et al. (2011) studied an isogenic daptomycin-susceptible and daptomycin-resistant pair of *S. aureus* strains (616 and 701) by using comparative proteomics of 616 vs. 701, revealing different concentrations of proteins in various functional categories, including cell wall-associated targets and biofilm formation proteins.

#### Colistin

Colistin is an antimicrobial peptide that interacts with the bacterial outer membrane, by displacing bacterial counter ions in the lipopolysaccharide (LPS). Hydrophobic/hydrophilic regions interact with the cytoplasmic membrane just like a detergent, solubilizing the membrane. The most common mechanisms of resistance to colistin are modifications to LPS (Bialvaei and Samadi Kafil, 2015).

Fernández-Reyes et al. (2009) induced resistance in the susceptible *A. baumannii* ATCC 19606 by growing the strain under increasing colistin pressure. These researchers then carried out 2-D difference gel electrophoresis (DIGE) experiments and identified 35 proteins that were expressed differently in the two phenotypes. Most of the proteins appearing were downregulated in the colistin-resistant strain. These include outer membrane (OM) proteins, chaperones, protein biosynthesis factors, and metabolic enzymes, indicating an important loss of biological fitness in the resistant phenotype. In a later study, Pournaras et al. (2014) sequentially collected samples from two colistin-susceptible/colistin-resistant (Col(s)/Col(r)) pairs of *A. baumannii* strains assigned to international clone 2, which is prevalent worldwide after extended exposure to colistin. These researchers observed underexpression of the protein CsuA/B and C from the csu operon in Col(r) isolate Ab347, relative to its Col(s) counterpart Ab299.

The biofilm efficiency of the *A. baumannii* 19606 type strain depends on the formation of pili, cell-surface appendages assembled via the CsuAB-A-B-C-D-E chaperone–usher secretion system, according to Tomaras et al. (2008). Chaperone usher (CU) pili are linear polymers made of subunits capable of either self-polymerization or assembly with other subunits. The biogenesis of CU fibers need a periplasmic chaperone and outer membrane assembly platform named the usher. Among the three chaperone systems, the archaic systems are associated with bacteria that cause some of the most serious diseases in humans, animals, and plants. This pilus is formed from four subunits, namely CsuA/B, CsuA, CsuB, and CsuE, and is assembled using the CsuC-CsuD chaperone-usher secretion machinery (Pakharukova et al., 2015).

In addition, the Col(r) isolate Ab347 underexpressed aconitase B and some enzymes implicated in the oxidative stress response, such as KatE catalase, superoxide dismutase, and alkyl hydroperoxide reductase. This possibly suggests a limited response to reactive oxygen species (ROS) and, therefore, impaired colistin-mediated cell death by means of hydroxyl radical production (Table 3).

**Table 3:** **Proteins involved in antibiotic resistance and virulence differentially expressed in Colr strain Ab347**.

**Accession no**.	**No. of peptides^a^**	**Score^b^**	**ANOVA^c^ (*P*)**	**Fold change^d^**	**Description**
ABYAL1639/katE	39	2919	1.78 × 10^−13^	−35.39	Catalase hydroperoxidase II
ABYAL3026	4	207	5.00 × 10^−15^	−28.34	Putative porin protein associated with imipenem resistance, CarO
ABYAL2670	6	549	3.27 × 10^−13^	−27.38	Putative protein CsuA/B
ABYAL 2667	3	130	1.24 × 10^−6^	−14.64	Putative protein CsuC
ABYAL2566/acnB	9	410	1.64 × 10^−9^	−3.13	Fragment of aconitate hydratase (part3)
ABYAL2567/acnB	7	332	1.43 × 10^−7^	−2.46	Fragment aconitate hydratase 2 (part 2)
ABYAL3715/sodC	7	376	1.41 × 10^−4^	−2.15	Superoxide dismutase precursor (Cu−Zn)
ABYAL1416/ahpC	14	881	1.81 × 10^−8^	−2.13	Alkyl hydroperoxide reductase (detoxification of hydroperoxides)
ABYAL2568/acnB	7	291	3.17 × 10^−6^	−2.12	Fragment of aconitate hydratase 2 (part 1)
ABYAL1790/*bla*_*OXA*−66_	3	104	2.17 × 10^−7^	−2.03	Carbapenem-hydrolyzing oxacillinase Oxa-66
ABYAL0009	9	578	1.08 × 10^−9^	+3.15	Putative RND-type efflux pump involved in aminoglycoside resistance (AdeT)

a *Number of peptides for identification and quantification*.

b *Confidence score for identification by Mascot software*.

c *ANOVA, analysis of variance*.

d *Negative values indicate underexpression in Col^r^ strain Ab347*.

*Obtained from Pournaras et al. (2014) and reprinted with permission from the publisher*.

The low intracellular c-di-GMP level in dispersed cells of a *P. aeruginosa* strain coincided with increased expression of proteins required for the virulence and development of antimicrobial peptide resistance in *P. aeruginosa* (Chua et al., 2013), and *P. aeruginosa* cells with low c-di-GMP levels were consequently found to be more resistant to colistin than *P. aeruginosa* cells with high c-di-GMP levels.

Chopra et al. (2013) analyzed the proteome of two strains of *A. baummanii*: the multidrug resistant *A. baummanii* strain BAA-1605 and the drug sensitive strain ATCC 1798. The analysis was performed by by iTRAQ labeling and online 2D LC/MS/MS for peptide/protein identification. A significant number of proteins were overexpressed at least twofold in the multidrug resistant strains including drug-, antibiotic-, and heavy metal-resistance proteins, porins, lipoproteins, stress-related proteins, membrane transporters, proteins important for acquisition of foreign DNA, cell-wall, and exopolysaccharide-related proteins, biofilm-related proteins, metabolic proteins, and many with no annotated function. However, the porin OmpW, less abundant in carbapenem- and colistin-resistant *A. baumannii* strains, was overexpressed by three times in BAA-1605.

### Conclusions

Greater knowledge of the specific mechanisms involved in bacterial resistance is needed to improve the treatment of diseases caused by infectious bacteria and thus control the survival of recalcitrant populations. This interesting area of research is complicated by the fact that multiple, super-imposed and/or balancing resistance mechanisms coexist in the same bacterial species.

Most proteomic analysis of antibiotic resistance can be classified into two broad groups: comparison of resistant and susceptible bacteria, and bacterial responses to the presence of antibiotics (Table 4). In general, in the first type of studies the least abundant proteins are related to secretion and metabolism, specifically OmpW, which is a known bacteriocin receptor. The most commonly expressed proteins are those involved in cell wall biogenesis, known resistance mechanisms, polysaccharide metabolism, and transport. The most frequently cited of these is Tolc, an outer membrane channel that participates in several efflux systems. In the second case, analysis of the bacterial response to antibiotic challenge revealed that the proteins most commonly affected are chaperone proteins and proteins involved in stress response, amino acid metabolism and energy metabolism. Some proteins involved in amino acid and energy metabolism are overexpressed while others are underexpressed, indicating the problem of using broad functional classifications and also the complexity of antibiotic response (Table 4). Some of the most revealing analysis coupled both previously described approaches, to investigate the response of both susceptible and resistant strains to antibiotic exposure. Multivariable approaches, i.e., those combining several strains, growth conditions, concentrations and types of antibiotics, and time points, may contribute to a better understanding of bacterial pathways and systems relevant to resistance.

**Table 4:** **Representative proteomic techniques for studying antibiotic resistance**.

**Proteomic technique (s)**	**Antibiotic**	**Physiological effects**	**Experimental approach**	**Pathogen (s)**	**References**
Two dimensional gel electrophoresis (2-DE) and tandem mass spectrometry	Penicillin	Growth phase, stress, and fatty acid biosynthesis (FAB) proteins expression altered	Wild type and *rgg* isogenic mutant with or without exposure to penicillin	*Streptococcus pyogenes*	Chaussee et al., 2006
2D-flurorescence difference gel electrophoresis (2D-DIGE)	Piperacillin/tazobactam	Bacterial virulence, antibiotic resistance, DNA protection, and multidrug efflux pump expression associated with resistance	Laboratory derived resistant strain and susceptible wild type	*Escherichia coli*	Dos Santos et al., 2010
iTRAQ	Oxacillin	LytR-CPsA-PsR (LCP) proteins, capsule, peptidoglycan biosynthesis, cell wall remodeling and urease proteins associated with oxacillin resistance	Methicillin resistant strain compared to isogenic oxacillin adapted strain	*Staphylococcus aureus*	Solis et al., 2014
2D-DIGE	Carbapenem	Beta-lactamases, energy, and protein production enzymes are upregulated; OmpW and surface antigen downregulated	Carbapenem resistant strains and wild type strain	*Acinetobacter baumannii*	Tiwari et al., 2012
2-DE and LC-MS/MS	Vancomycin	Vancomycin resistance proteins upregulated; metabolism-related proteins downregulated	Vancomycin resistant strain with or without exposure to vancomycin	*Enterococcus faecium*	Ramos et al., 2015
SDS-PAGE electrophoresis and LC-MS/MS	Chloramphenicol	Overexpression of efflux pump systems associated with resistance	Laboratory derived resistant strain and susceptible wild type	*Burkholderia thailandensis*	Biot et al., 2011
2-DE and iTRAQ	Linezolid	Metabolism and transport of carbohydrates involved in resistance to linezolid	Linezolid resistant and wild type strains	*Streptococcus pneumoniae*	Feng et al., 2011
2-DE/MS-MS and 1-DE /LC/MS-MS	Tetracycline	Outer membrane proteins decreased expression in membrane and increased secretion	Response to sub-minimal inhibitory concentrations of tetracycline	*Acinetobacter baumannii*	Yun et al., 2008
2-DE and LC-MS/MS	Kanamycin	Outer membrane protein expression altered. Identification of novel membrane MipA protein involved in antibiotic resistance	Laboratory derived resistant and susceptible strains	*Escherichia coli*	Li H. et al., 2015
2-DE and LC-MS/MS	Erythromycin	Glyceraldehyde-3-phosphate dehydrogenase upregulation in resistant strain	Susceptible and resistant strains	*Streptococcus pneumoniae*	Cash et al., 1999
iTRAQ and 2D-LC-MS/MS	Metronidazole	RecA, ferric uptake regulator (Fur), putative nitroreductases and altered expression of stress-related proteins	Susceptible and resistant strains before and after treatment with antibiotic	*Clostridium difficile*	Chong et al., 2014
2-DE and LC-MS/MS	Fluoroquinolone (ciprofloxacin)	Overexpression of ATP-binding component of ATP binding cassette (ABC)	Laboratory derived resistant and susceptible strains	*Pseudomonas aeruginosa*	Zhou et al., 2006
2-DE and LC-MS/MS	Rifampicin	Alterations in several metabolic processes and secretion mechanisms	Laboratory derived resistant and susceptible strains	*Brucella abortus*	Sandalakis et al., 2012
iTRAQ and IPG-isolectric focusing with LC-MS	Daptomycin	Differences in biofilm formation proteins, cell wall-associated targets	Isogenic daptomycin susceptible and resistant strain pair	*Staphylococcus aureus*	Fischer et al., 2011
2-DE and LC-MS/MS	Colistin	Outer membrane proteins, chaperones, protein biosynthesis factors and metabolic enzymes downregulated associated with loss of biological fitness	Laboratory derived resistant and susceptible strains	*Acinetobacter baumannii*	Fernández-Reyes et al., 2009

Some unique mechanisms or groups of proteins are expressed at low or indiscernible levels in the unchallenged resistant organisms, but are regulated in response to antibiotic exposure. These represent potentially useful targets in new therapies against resistance.

## Analysis of antimicrobial resistance for diagnostic purposes

### General aspects

The main purpose of a clinical microbiology laboratory is to provide reliable information as quickly as possible about the etiological agents responsible for infections and their sensitivity to antibiotics. The information obtained should allow enable selection of the most appropriate antimicrobial therapy to improve the care provided to the patient while at the same time contributing to better control of resistance and also to decreasing expenditure on antibiotics (DeMarco and Ford, 2013).

Recent findings indicate the potential usefulness of mass spectrometry techniques, in particular MALDI-TOF MS, to identify specific mechanisms of resistance. These techniques are less expensive and produce results more quickly than the currently used methods. The applications of MALDI-TOF MS for diagnosis of antibiotic resistance have been discussed in two recent excellent reviews (Hrabák et al., 2013; Kostrzewa et al., 2013). These methods are at the initial stages of evaluation and some are only useful for centers of excellence and research laboratories.

Here, we will try to summarize and classify methods available with some valuable examples and providing the latest contributions. MALDI-TOF MS applications in detection of antimicrobial resistance can be classified depending on the type of target and the methodology into the following approaches.

### Identification of the entire cell profile

Identification of the entire cell profile involves establishing differences in the whole protein spectra profile of susceptible and resistant strains. This approach has been used to identify methicillin-resistant *S. aureus* (MRSA), with the first attempts being carried out by Edwards-Jones et al. (2000) at Manchester University. MRSA and MSSA strains were subsequently successfully distinguished using a protein Chip array (SELDI-TOF, Surface-enhanced laser desorption/ionization; Shah et al., 2011). Retrospective typing of an MRSA outbreak showed that it is possible to differentiate unrelated MSSA, MRSA and borderline resistant *S. aureus* (BORSA) strains.

MALDI-TOF MS has been also used to detect vancomycin-resistant enterococci. The method was used to detect vanB positive *Enterococcus faecium* with a high sensitivity and specificity (Griffin et al., 2012), and vanA positive *E. faecium* has recently been detected (Nakano et al., 2014). Similar findings have recently been reported for an important Gram-negative anaerobic pathogen, *B. fragilis*. Two groups (division I and II), division II harboring the gene cfiA, encoding a potent metallo-beta-lactamase have been differentiated by a specific peak in their MALDI-TOF profile spectra (Nagy et al., 2011; Wybo et al., 2011).

MALDI-TOF MS techniques have also been used by different researchers to differentiate beta-lactamase genes carrying strains of *Enterobacteriaceae* and *P. aeruginosa*. Dubska et al. (2011) used SELDI-TOF MS, but were unable to clearly differentiate different beta-lactamases. However, Camara and Hays (2007) detected a peak corresponding to a beta-lactamase in *E. coli* strains. Overall the results suggest that the routine detection of beta-lactamase producing strains is not yet possible in clinical laboratories.

### Identification of an antibiotic and product of hydrolysis

This promising approach has been reported for the important clinical carbapenemases and extended-spectrum beta-lactamases. The mass shift that occurs during the addition of a water residue in the beta-lactam molecule after enzymatic hydrolysis can be detected by MALDI-TOF MS. The beta-lactamase negative strains do not modify the molecular weight of the beta-lactam (Hrabák et al., 2013; Kostrzewa et al., 2013). Moreover, the procedure enables quantitative analysis that is useful for direct comparison with MIC-values, and it also provides rapid resolution. Furthermore, the method can be improved by using beta-lactamase inhibitors to identify specific types of beta-lactamase and it is applicable to positive blood cultures. The main limitation is the interference with other resistance mechanisms such as porins and efflux pumps when intact cells are used; however, this can be overcome by using different lysis methods. Analysis of the raw spectra can also be difficult for unexperienced microbiologists.

**Figure 1:**
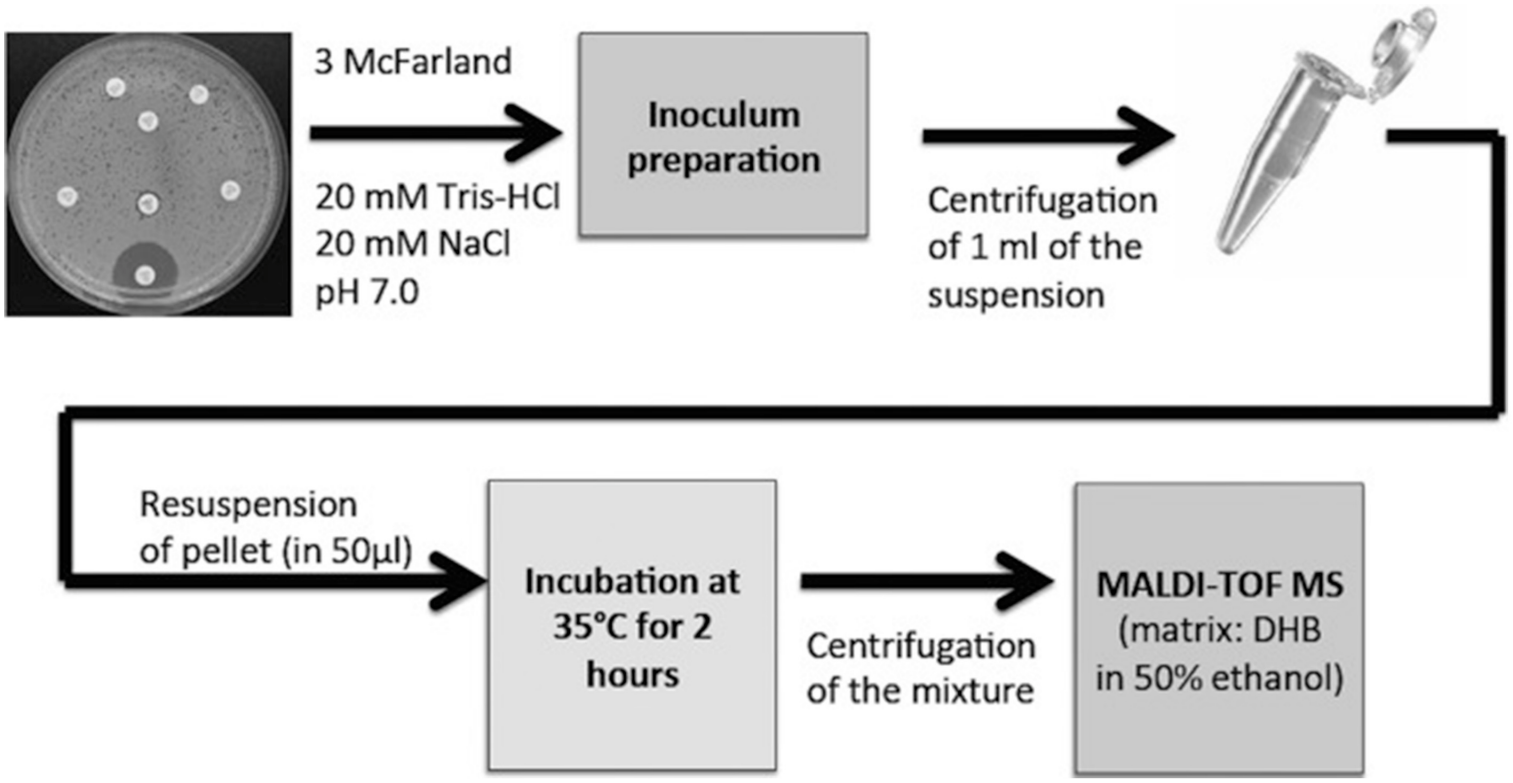
**Basic protocol for detecting beta-lactam hydrolyzed products by MALDI-TOF MS**. 3 McFarland is a measure of optical density of the bacterial culture. Obtained from Hrabák et al. (2013) and reprinted with permission from the publisher.

The first two studies involving direct carbapenemase detection were both published in 2011 (Burckhardt and Zimmermann, 2011; Hrabák et al., 2011). The prevailing approach consists of the incubation of a pellet from a bacterial culture, usually grown overnight, with the beta-lactam antibiotic. After incubation of the culture for 1–3 h at 35°C, the supernatant is analyzed by MALDI-TOF MS (Figure 1). The addition of NH_4_HCO_3_ increased the sensitivity of detection of OXA-48 (Papagiannitsis et al., 2015; Studentova et al., 2015). The method has also been extended to OXA-51 and SIM-1 carbapenemases (Lee et al., 2013). MALDI-TOF MS can be used detect OXA- and GES-carbapenemases (Chong et al., 2015). The method has been used to detect carbapenemase resistance associated with cifA encoded metallo-beta-lactamase in blood samples (Johansson et al., 2014). The capacity of these techniques for detecting carbapenemases has recently been enhanced by different research groups (Álvarez-Buylla et al., 2013; Peaper et al., 2013; Wang et al., 2013). The method has been shown to be useful in detecting ESBL-producing *Enterobacteriaceae* from positive blood cultures, with 99% of sensitivity within a maximum of 150 min, with cefotaxime and ceftazime antibiotics (Oviaño et al., 2014).

Some MALDI-TOF MS based methods can also be used to detect modifications that occur in resistance to aminoglycosides, such as inactivation by acetyltransferases (Kostrzewa et al., 2013), with poor reproducibility and rRNA methyltransferase (Hrabák et al., 2013). Overall these data suggest that MALDI-TOF can be used to detect aminoglycoside resistance, although these methods are generally limited to research laboratories.

### Detection of resistance proteins within the cell

In this case, MALDI-TOF MS can be used to help detect some microbial biomarkers (essentially proteins or their fragments, obtained after trypsin digestion) that confer resistance to the pathogen.

For example, methicillin-resistant *S. aureus* positive for agr (accessory gene regulator) and harboring the class A mec complex was identified by detection of the small peptide called PSM-mec in whole cells (Josten et al., 2014).

**Figure 2:**
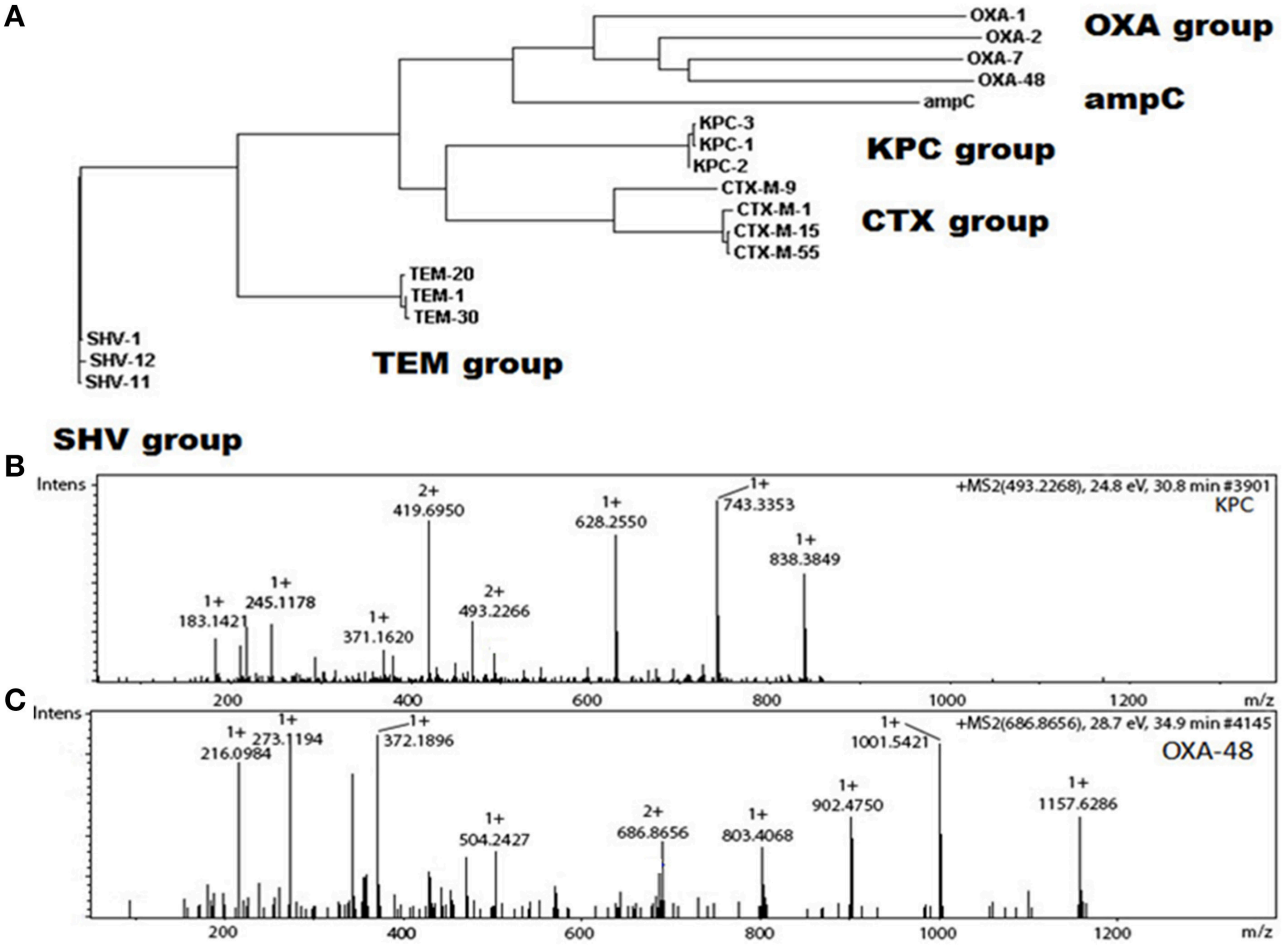
**Differentiation between different β-lactamase subgroups and species. (A)** Phylogenetic tree based on a sequence alignment of different β-lactamases from different functional subgroups. For some classes, the primary structures are very similar (e.g., KPC and CTX) while for others, individual sequences vary considerably (e.g., OXA). **(B)** Tandem mass spectrum for a unique tryptic peptide from the KPC-group of carbapenemases (FPLCSSFK). **(C)** Tandem mass spectrum for a unique tryptic peptide from OXA-48 (SQGVVVLWNENK). Obtained and adapted from Fleurbaaij et al. (2014) and reprinted with permission from the publisher.

Detection of antibiotic resistance markers, such as peptide fragments, in clinical strains of *E. coli* associated with some beta-lactamases (CTX-M1, CMY-2, VIM, and TEM) together with kanR and aminoglycoside modifying enzyme was obtained by periplasm extraction followed by nano-LC separation (Hart et al., 2015) Capillary-electrophoresis mass spectrometry is useful for detecting OXA-48 and KPC carbapenemases in multi-drug resistant Gram-negative bacteria (Fleurbaaij et al., 2014; Figure 2), and detection of beta-lactamase proteins has been improved by the use of MALDI-TOF MS methods rather than PCR detection of the corresponding genes (Trip et al., 2015).

### Cell wall analysis

The cell wall is the target of most antibiotics and is a barrier to other antibiotics that act in the cytosol. Some components of the outer membrane of Gram-negative bacteria such as porins, efflux pumps, and lipopolysaccharides have been quantified by MALDI-TOF MS techniques to distinguish between resistant and sensitive strains (Peng et al., 2005; Imperi et al., 2009). For example, a MALDI-TOF MS based method proved better than SDS-PAGE for characterizing the Ompk36 porin in *K. pneumoniae* (Cai et al., 2012). Changes in the structure of the lipopolysaccharide lipid A that occur during emergence of resistance to colistin can be detected by MALDI-TOF MS in *A. baumanni* (Beceiro et al., 2011).

### Discovery of mutations within resistance genes through mini-sequencing

**Figure 3:**
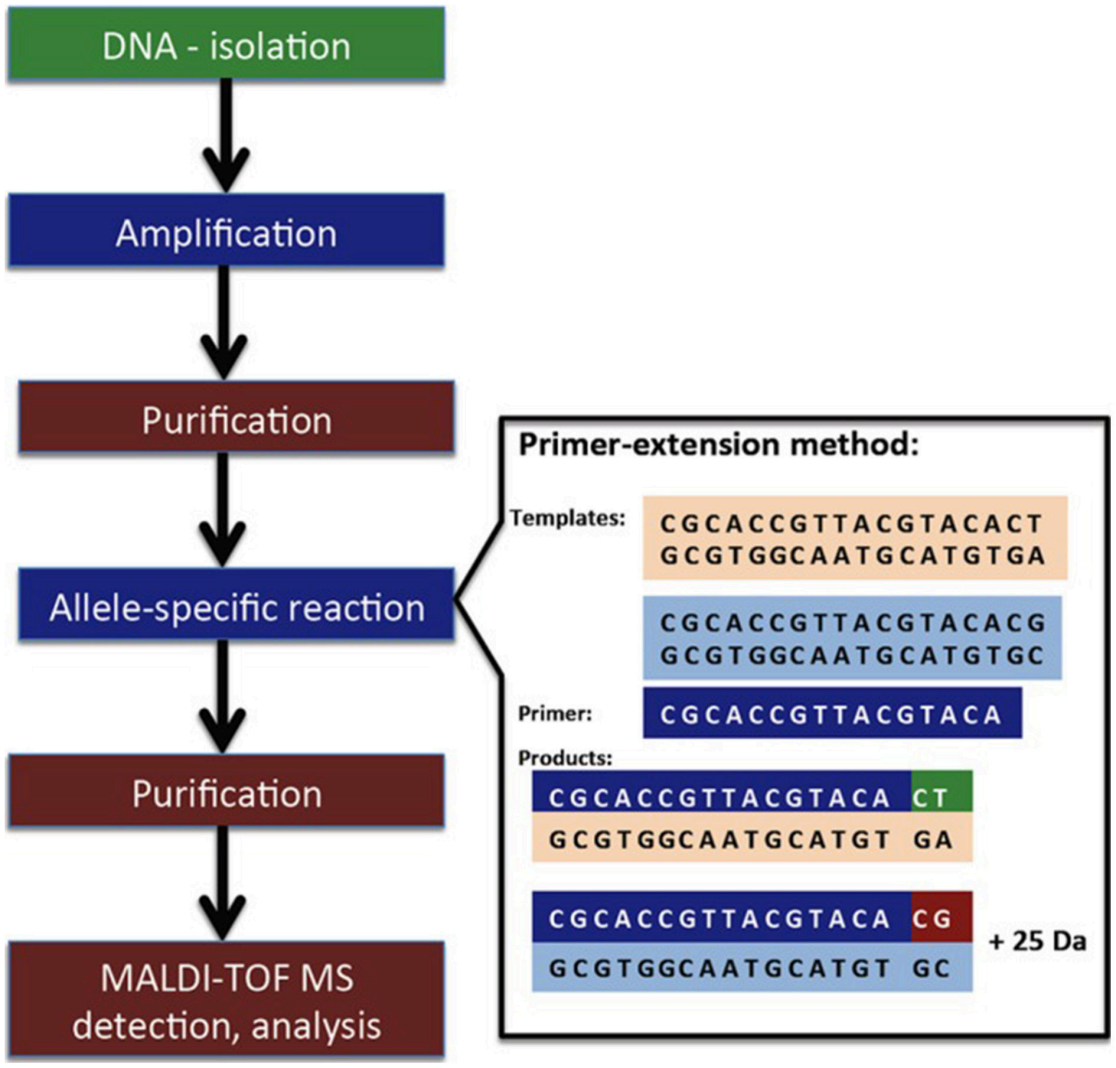
**Simplified procedure of minisequencing linked to MALDI-TOF MS detection**. Obtained from Hrabák et al. (2013) and reprinted with permission from the publisher.

MALDI-TOF MS methods have been used to analyse DNA sequencing (Pusch et al., 2002). The technique is based on a primer extension assay for only a few bases, and the molecular weight shift is then detected by MALDI-TOF MS. The technique is limited to DNA molecules smaller than 40 bp (Figure 3).

Although, it has been used to detect mutations for a large and diverse number of resistance mechanism (Hrabák et al., 2013), this approach is time-consuming and does not confer any advantages over standard sequencing protocols.

PCR/electrospray ionization Mass spectrometry (PCR/ESI MS), which involves mini-sequencing of larger PCR products (110–450 bp), has been successfully used to identify pathogens and has also proved useful for detecting some *bla*_KPC_ beta-lactamase producing Enterobacteria (Endimiani et al., 2010). This technique has been commercialized via development of a fully automated system (PLEX-ID, Abbot Biosciencies).

### Stable isotope labeling and monitoring of cell growth

The technique has been used for monitoring resistance to fluconazole (Marinach et al., 2009) and caspofungin (De Carolis et al., 2012) in fungi. Cells harvested from solid cultures were incubated with different concentrations of antibiotic for 24 h and the total peak profile was determined; however, no improvement in time-to-result was achieved, despite the good correlation between MIC-values and different spectra. A simplified version of this approach that facilitates discrimination of susceptible and resistant isolates of *Candida albicans* after incubation for 3 h in the presence of “breakpoint” level drug concentrations of the caspofungin has been described (Vella et al., 2013).

Some studies have attempted to detect bacterial resistance by incorporating stable isotope labeled nutrients during growth in the presence of antibiotics (Demirev et al., 2013). The peak profiles were compared with those of intact microorganisms grown in unlabeled media without the drug. Some biomarkers were detected by characteristic mass shifts provided by a suitable isotope. A similar approach has recently been used with ^13^C${}_{6}^{15}$N_2_-L-lysine as the isotope for detection of methicillin-resistant *S. aureus* (Sparbier et al., 2013). Stable isotope labeling by amino acids in cell culture (SILAC) technology with the same isotope have been used to distinguish resistant and susceptible strains of *P. aeruginosa* to meropenem, tobramycin, and ciprofloxacin (Jung et al., 2014).

### Conclusions

Although, the use of MALDI-TOF MS in clinical microbiology has been successfully applied in identifying bacteria, detection of antibiotic resistance, essential in bacterial diagnostics, is still at an early stage of development. Some promising achievements are the detection of proteins that hydrolyze antibiotics by means of direct detection of antibiotic modifications. The most remarkable example is the detection of beta-lactamases, particularly carbapenemases, by a method that has been validated and routinely used in clinical and reference laboratories. However, detection of resistance mechanism elements (particularly Qnr proteins and mutant PBP and beta-lactamases) for improved diagnosis of multiresistant bacteria must be improved and validated, despite some reports about the detection of vancomycin resistant *Enterococcus* spp. and discrimination between MRSA and MSSA strains.

In summary, several of the cited methods require further validation, simplification and automation. MALDI TOF-MS analysis may prove to be too labor intensive for routine use in clinical laboratories. Other challenges include the better study of the interaction between proteomic results and MIC-values for used in prescribing appropriate treatment and monitoring infection. Methods of testing multiple antibiotics are urgently required in order to provide a complete picture of antibiotic resistance. Standard susceptibility testing will probably coexist with new proteomic methods, as resistance to antibiotic is a complex process.

## Proteomics for studying bacterial virulence

### General aspects

Proteomic techniques are becoming important tools for investigating microbial pathogenesis. Applications include identification of virulence factors and study of the response of both host and pathogen to infection.

Research was initially limited to the classical proteomic analysis of protein contents of bacterial cultures, usually involving comparison of avirulent and virulent strains or conditions that simulate stresses that bacteria encounter during the infection process, such as acid stress, low oxygen content, high osmolarity, and other conditions of temperature, presence or limited availability of iron and presence of urine or plasma. Co-cultivation with host cell cultures and the direct isolation of bacteria from samples were not achieved until a later stage, because of the technical challenge that arose in measuring bacterial proteins against the overwhelming background of host proteins. Different approaches have been used to solve these problems, usually involving different separation techniques such as differential centrifugation, cell sorting, and immunomagnetic separation. Some review articles have already considered these early studies (Bhavsar et al., 2010; Cash, 2011; Yang et al., 2015), and we therefore focus on recent studies that we consider important in the study of bacterial pathogenesis by proteomics.

### Bacterial pathogenesis

Wang et al. (2014) recently compared the proteome profile of the *S. enterica* subsp. *enterica* serovar *Typhimurium* and *S. typhi*, which are responsible for, respectively, gastroenteritis and typhoid fever types. These researchers first detected a group of proteins with serovar-specific expression, which can be used as new biomarkers for identifying clinical serotypes. They also found that expression of flagella and chemotaxis proteins was lower in *S. typhi* than in *S. typhimurium*. Finally, the expression of core genes, which were involved in metabolism and transport of carbohydrates and amino acids, differed in the two serovars.

Proteomics, microbial genetics, competitive infections, and computational approaches have previously been used to obtain a comprehensive overview of *Salmonella* nutrition and growth in a mouse typhoid fever model (Steeb et al., 2013). In a study of the virulence of *H. pylori*, Vitoriano et al. (2011) compared the proteomes of strains of the bacterium isolated from children with peptic ulcer disease and from children with non-ulcer dyspepsia. In addition to the presence of genes clearly encoding virulence factor, the pediatric ulcerogenic strains presented a proteome profile defined by changes of motility-associated proteins (involved in higher motility), antioxidant proteins (involved in better resistance to inflammation), and proteins implicated in key functions in the metabolism of glucose, amino acids, and urea (probably advantageous for confronting changes in nutrient availability).

More recently, Ansong et al. (2013a) performed a temporal multi-omic analysis, at physiologically relevant temperatures, of *Yersinia pestis* (YP), the causative agent of plague with a high mortality rate, and *Yersinia pseudotuberculosis* (YPT), an enteric pathogen with a modest mortality rate. Gene and protein expression levels of conserved major virulence factors were higher in YP than in YPT, including the Yop virulon and the pH6 antigen. The global transcriptome and proteome responses of YP and YPT revealed conserved post-transcriptional control of metabolism and the translational machinery including the modulation of glutamate levels in *Yersiniae*.

Madeira et al. (2013) compared the proteomic profiles of three clonal isolates of *Burkholderia cenocepacia* obtained from a cystic fibrosis patient between onset of infection and before death from cepacia syndrome 3.5 years later. They found that 52 proteins were similarly altered in both late-stage isolates, relative to the first isolate, which suggests an important role for metabolic reprogramming in the virulence potential and persistence of *B. cenocepaci*a, particularly in regard to bacterial adaptation to microaerophilic conditions. The content of the virulence determinant AidA was also higher in the two late-stage isolates.

In an original approach, Provenzano et al. (2013) analyzed the metaproteome of microbial communities from endodontic infections associated with acute apical abscesses and asymptomatic apical periodontal lesions. They found several proteins related to pathogenicity and resistance/survival in endodontic samples, including proteins involved in adhesion, biofilm formation, and antibiotic resistance, stress proteins, exotoxins, invasins, proteases, and endopeptidases (mostly in abscesses), and an archaeal protein linked to methane production. Most of the human proteins detected were involved in cellular processes and metabolism, as well as immune defense.

In a study involving quantitative proteomic analysis, Liu et al. (2012) showed that on entry into host cells, *Campylobacter jejuni* undergoes a significant metabolic downshift. They also observed reprogramming of respiration in intracellular *C. jejuni*, favoring respiration of fumarate.

Pieper et al. (2013) performed global proteomic analysis of *Shigella flexneri* strain 2457T in association with three distinct growth environments: in broth (*in vitro*), in epithelial cell cytoplasm (intracellular), and coculture with extracellular epithelial cells. The intracellular bacteria showed elevated protein expression in invasion and cell-to-cell spread determinants, including IpA, Mxi, and Ics proteins, compared with *in vitro* and extracellular bacteria. The intracellular environment was also characterized by changes in iron stress and carbon metabolism protein expression levels, which may indicate its important role in the transition of *S. flexneri* from the extra to the intracellular milieu.

Another elegant study identified the pathogenic mechanisms of *Chlamydia trachomatis* after entry into host cells and the formation of a membrane-bound compartment (the inclusion) accompanied by secretion of inclusion membrane proteins (Incs). Mirrashidi et al. (2015) used affinity purification-mass spectroscopy (AP-MS) to detect Inc-human interactions for 38/58 Incs involved in host processes of intracellular life cycles, including retromer components as sorting nexins. Inc targets and overlapping of viral proteins were detected, suggesting common pathogenic mechanisms among obligate intracellular microbes.

**Figure 4:**
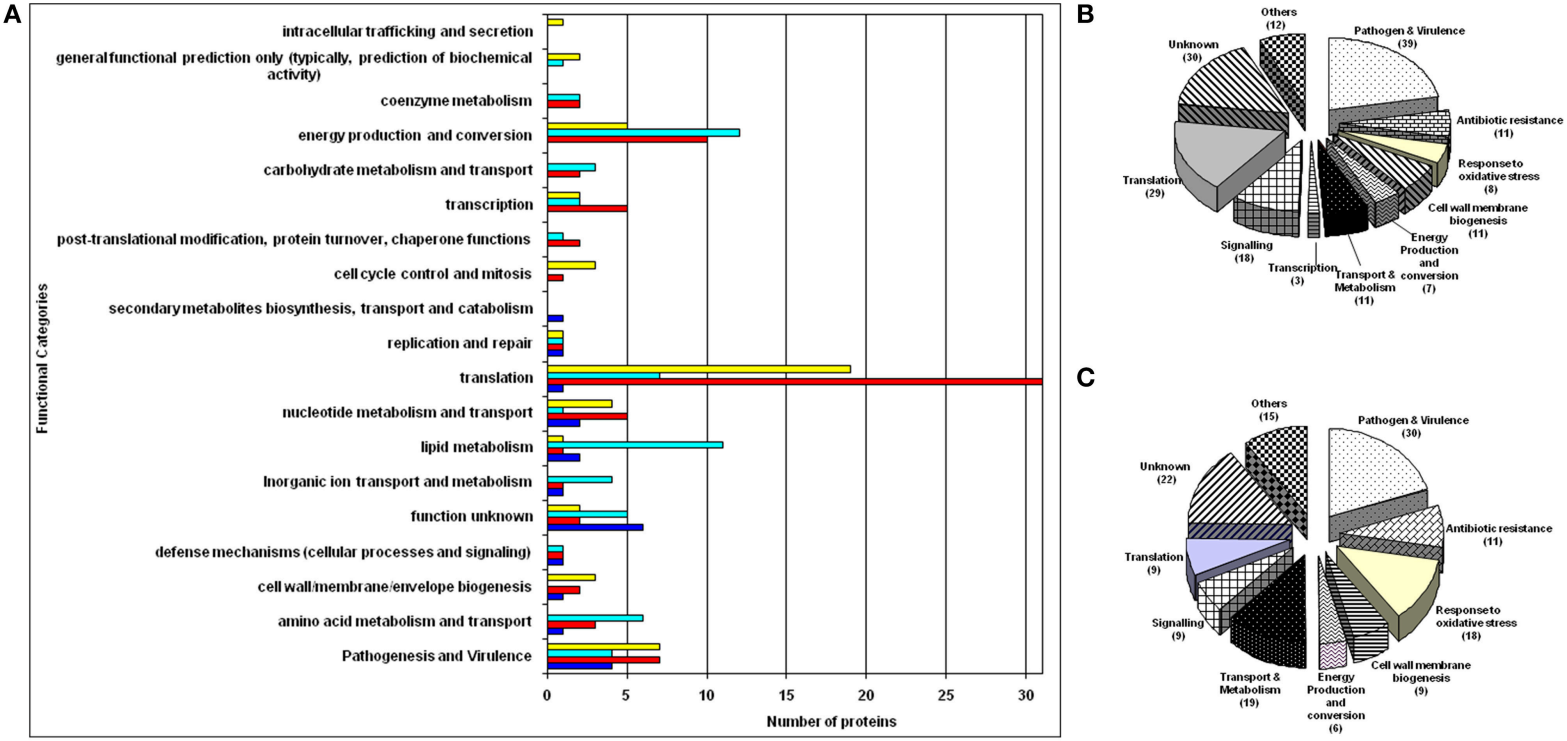
**(A)** Distribution of differently expressed proteins in *A. baumannii* following *ex vivo* incubation according to functional categories. The stacked bar chart shows the number of overexpressed proteins (red) and underexpressed proteins (dark blue) in the macrophage model and the number of overexpressed proteins (yellow) and underexpressed proteins (light blue) in the bronchoalveolar lavage fluid (BALF) model in each functional category. Obtained and adapted from Méndez et al. (2015) and reprinted with permission from the publisher. Functional classification of the proteins identified in the *A. baumannii* extracellular sub-proteomes. **(B)** Proteins from Outer Membrane Vesicle subproteome and **(C)** Freely soluble extracellular proteins. The total numbers of proteins within the respective group are shown in brackets. Obtained and adapted from Mendez et al. (2012) and reprinted with permission from the publisher.

During a very recent study with the infectious agent *A. baumannii*, we obtained an *ex vivo* protein expression profiling for pneumonia (Méndez et al., 2015). We characterized the proteome of *A. baumannii* in the presence of bronchoalveolar lavage fluid from infected rats (to simulate conditions in the respiratory tract) and in the presence RAW 264.7 cells (control conditions). We observed alterations in cell wall synthesis and identified two upregulated virulence-associated proteins with >15 peptides/protein in both *ex vivo* models (OmpA and YjjK), which suggests that these proteins are fundamental for pathogenesis and virulence in the airways (Figure 4A).

### Studies of microbial biofilm

At least 65% of all human infectious diseases are known to be associated with the biofilm form of microorganisms. The most notable and clinically relevant property of biofilms is the greater resistance to antimicrobials than shown by their planktonic counterparts (Seneviratne et al., 2012). Some proteomic studies have revealed an association between resistance mechanisms and biofilm state, the subject of a recent review article (Seneviratne et al., 2012). Our research group has been able to identify a putative resistance-nodulation-cell division type efflux pump (RND pump), which increased in *A. baumannii* biofilms. Overexpression of this type of efflux pump in *A. baumannii* confers resistance to aminoglycosides, as well as to other drugs, including fluoroquinolones, tetracyclines, chloramphenicol, erythromycin, trimethoprim, and ethidium bromide (Cabral et al., 2011). In another study recently carried out in our laboratory, *A. baumannii* clinical strain AbH12O-A2 was able to survive long periods of desiccation as a result of the presence of cells in a dormant state, via mechanisms affecting control of cell cycling, DNA coiling, transcriptional and translational regulation, protein stabilization, antimicrobial resistance, and toxin synthesis; a few surviving cells embedded in a biofilm matrix were able to resume growth and restore the original population in appropriate environmental conditions (Gayoso et al., 2014).

Relationships between biofilm state and virulence factors have also been discovered recently. High-resolution 2-dimensional gel electrophoresis was used to obtain the proteome profiles of spiral *H. pylori* and early biofilm (Shao et al., 2013). Differential protein spots were identified and associated with flagellar movement, bacterial virulence, signal transduction, and regulation.

MALDI-TOF MS/MS analysis of the secretome of single and mixed biofilms of *P. aeruginosa* and *C. albicans* carried out at different times (Purschke et al., 2012) identified 16 proteins overexpressed or exclusively expressed in *P. aeruginosa* when interacting with *C. albicans* including virulence factors such as exotoxin A and iron acquisition systems.

Another strategy was used to study the biofilm expression profile in a clinical strain of *S. pneumoniae* serotype 14. A decrease in the expression of enzymes related to the glycolytic pathway, as well as proteins involved in translation, transcription, and virulence was detected during biofilm growth by the proteomics method (Allan et al., 2014).

### Outer membrane vesicles and their impact on virulence

Outer membrane vesicles (OMVs) are nanoscale structures secreted by bacteria and that can carry nucleic acids, proteins, and small metabolites. They mediate intracellular communication and play a role in virulence. One of the early proteomic studies of the protein fraction of the outer membrane vesicles was carried out in our laboratory. This study focused on two main protein fractions of the extracellular proteome: proteins exported by outer membrane vesicles (OMVs) and freely soluble extracellular proteins (FSEPs) present in the culture medium of a highly invasive, multidrug-resistant strain of *A. baumannii* (clone AbH12O-A2; Mendez et al., 2012). Of the OMV proteins, 39 were associated with pathogenesis and virulence, including proteins associated with attachment to host cells (e.g., CsuE, CsuB, CsuA/B) and specialized secretion systems for delivery of virulence factors (e.g., P-pilus assembly and FilF), whereas the FSEP fraction possessed extracellular enzymes with degradative activity, such as alkaline metalloprotease. Among the FSEP, we have also detected at least 18 proteins with a known role in the oxidative stress response (e.g., catalase, thioredoxin, oxidoreductase, superoxide dismutase; Figures 4B,C). These findings are supported by those of a recent study of other clinical *A. baummanni* strains in which it was discovered that *A. baumanni* strain A38 possesses an outer membrane vesicle containing virulence factors including Omp38, EpsA, Ptk, GroEL, hemagglutinin-like protein, and FilF (Li Z. T. et al., 2015). The high content of virulence factors in outer membrane vesicles has been demonstrated in other Gram-negative bacteria such as clinical strains of *E. coli* and *Stenotrophomonas maltophila* (Devos et al., 2015; Wurpel et al., 2015). Indeed, this phenomenon is also conserved in some Gram-positive pathogenic strains such as *M. tuberculosis* and *S. pneumoniae* (Olaya-Abril et al., 2014; Lee J. et al., 2015).

However, as pointed out by several authors (Koning et al., 2013; Pérez-Cruz et al., 2013), OMVs may be composed by inner membrane fractions and can thus be considered outer-inner membrane vesicles. These structures may thus contain cytosolic fractions. OMV-related virulence can be considered in the context of this novel concept.

### Post-translational protein modifications and their impact on virulence

Bacteria contain different types of post-translational modifications, many of which are commonly present in eukaryotes: phosphorylation, acetylation, glycosylation, ubiquitination, and glutathionylation (Soufi et al., 2012). Relationships between these types of post-translational modifications and virulence have been established in recent years. One of the first studies with *Pseudomonas* species revealed that phosphoproteins involved in motility, transport and pathogenicity pathways in *P. aeruginosa* are critical for survival and virulence (Ravichandran et al., 2009).

Around the same time, the phosphoproteome of *K. pneumoniae* NTUH-K2044 was analyzed by a shotgun approach, and 117 unique phosphopeptides were identified along with 93 *in vivo* phosphorylated sites corresponding to 81 proteins (Lin et al., 2009). Interestingly, three of these were found to be distributed in proteins of the cps locus and the authors of the study speculated that they were involved in the converging signal transduction of capsule biosynthesis, which has been related to virulence. Involvement of phosphorylated proteins in virulence was also hypothesized after proteomic analysis of *S. pneumoniae* and *Mycoplasma pneumoniae* (Schmidl et al., 2010; Sun et al., 2010).

Interestingly, qualitative comparison between the Ser/Thr/Tyr phosphoproteomes of two *A. baumannii* strains (a reference strain and a highly invasive multidrug-resistant clinical isolate) led to the identification of phosphoproteins with a role in pathogenicity and also involved in drug resistance (Soares et al., 2014).

Other types of post-translational modifications have recently been discovered by proteomic methods. In a well-designed study, Iwashkiw et al. (2012) showed that *A. baumannii* ATCC 17978 possesses an O-glycosylation system responsible for the glycosylation of multiple proteins. These authors identified seven *A. baumannii* glycoproteins, of yet unknown function, by 2D-DIGE and mass spectrometry. A glycosylation-deficient strain was generated by homologous recombination. This strain did not show any growth defects, but exhibited a severely diminished capacity to generate biofilms. Disruption of the glycosylation machinery also resulted in reduced virulence in two infection models (*Dictyostelium discoideum* and *Galleria mellonella*) and reduced *in vivo* fitness in a mouse model of peritoneal sepsis. In a study involving proteomic and glycoproteomic analysis, Scott et al. (2014) found higher N-linked glycosylation in *Campylobacter jejuni* NCTC11168 O clinical strain than in the laboratory-derived strain.

Another post-translational modification was described by Xie et al. (2015) who investigated proteome lysine acetylation profiling in *M. tuberculosis* by using a combination of anti-acetyl lysine antibody-based immunoaffinity enrichment and high-resolution mass spectrometry. These authors identified 1128 acetylation sites on 658 acetylated *M. tuberculosis* proteins, several of which, e.g., isocitrate lyase, were involved in the persistence, virulence and antibiotic resistance. In an interesting study, Ansong et al. (2013b) identified different uses for the protein S-thiolation forms S-glutathionylation and S-cysteinylation in response to infection-like conditions and basal conditions in *S. typhimurium* and supported by analysis of protein structure and gene deletion.

### Conclusions

The use of proteomic analysis to study the interactions between bacterial pathogenesis and host is at a very early stage. Nevertheless, we have described the use of proteomic techniques to investigate many aspects of bacterial pathogenesis: assignment of a virulence factor reservoir of a single pathogen, analysis of the interaction between host and pathogen response during the infection, identification of interactions between several virulence factors and host cell components and even the identification of biochemical action of virulence factors (Table 5).

**Table 5:** **Representative proteomic techniques for studying virulence determinants**.

**Proteomic technique (s)**	**Physiological effects**	**Experimental approach**	**Pathogen (s)**	**References**
SDS-PAGE electrophoresis and LC-MS/MS	Metabolic downshift, reprogramming of respiration to fumarate	Isolation of bacteria from intracellular culture	*Campylobacter jejuni*	Liu et al., 2012
LTQ-Orbitrap Velos and LC-QTOF analysis	Identification of proteins involved in adhesion, biofilm formation, antibiotic resistance, stress proteins, exotoxins, invasions, proteases, and endopeptidases	Metaproteome analysis	Endodontic bacteria	Provenzano et al., 2013
2-D DIGE	Metabolic reprogramming to microaerophilic conditions, upregulation of vilrulence determinant AidA, and iron uptake proteins	Study of clonal isolates in different stages during cystic fibrosis infection	*Burkholderia cenocpacia*	Madeira et al., 2013
2-DE and LC-MS/MS	Yop virulon and pH 6 antigen overexpressed in *Yersinia pestis*. Modulation of glutamate levels.	Proteomic analysis at physiologically relevant temperatures	*Yersinia pestis* and *Yersinia pseudotuberculosis*	Ansong et al., 2013a
2-DE and LC-MS/MS	Ulcerogenic strains associated with virulence factors, motility proteins, antioxidant proteins, and metabolism of glucose, amino acids, and higher expression of urea proteins	Comparision of clinical isolates with peptic ulcer disease with non-ulcer dyspepsia	*Helicobacter pylori*	Vitoriano et al., 2011
iBAQ label free	Reprogramming of metabolism	Bacteria isolated from mouse typhoid fever model	*Salmonella enterica*	Steeb et al., 2013
SILAC	Flagella and chemotaxis proteins downregulated in *Salmonella typhi*.	*In vitro* cultures of both strains	*Salmonella typhimurium* and *Salmonella typhi*	Wang et al., 2014
	Identification of strain associated biomarkers			
Affinity purification-mass spectroscopy (AP-MS)	Identification of interactions of inclusion membrane proteins with sorting nexins, components of the retromer.	Human targets of bacterial inclusion membrane proteins	*Chlamydia trachomatis*	Mirrashidi et al., 2015
iTRAQ and LC-MS/MS	Alterations in cell wall synthesis and upregulation of virulence proteins OmpA and Yjjk	Response to host airways “*ex vivo*” models	*Acinetobacter baumannii*	Méndez et al., 2015
2-DE and LC-MS/MS	Biofilm associated proteins related to flagellar movement, bacterial virulence, and signal transduction and regulation	Biofilm analysis	*Helicobacter pylori*	Shao et al., 2013
iTRAQ	Decrease in proteins involved in glycolytic, translation, transcription, and virulence. Increase in proteins, pyruvate, carbohydrate, and arginine metabolism	Biofilm analysis	*Streptococcus pneumoniae*	Allan et al., 2014
2-DE and LC-MS/MS	Intracellular bacteria increased expression of invasion and cell-to-cell spread proteins. Mixed-acid fermentation pathway proteins and iron stress proteins upregulated in intracellular bacteria	Comparison of *in vitro*, intracellular and extracellular cultures	*Shigella flexneri*	Pieper et al., 2013

The limit of sensitivity has prevented a more detailed proteomic study of *in vivo* models of infection, particularly in humans, in comparison with tissue culture models. Although, current methods of analysis are proving very useful, they must be improved further in order to advance our knowledge in this field. A multidisciplinary approach including methods such as immmunoassays, histological methods, and electron microscopy should be undertaken for an integrated understanding of bacterial pathogenesis. Monitoring of the temporal and spatial action of virulence factors may also contribute to advances in this field.

The relationships between biofilm formation, outer membrane vesicles, and virulence have been highlighted during this review.

Identification of variations in post-translational changes in the bacteria and/or in the host proteomes after infection is also important for studying interactions between signaling cascades.

A detailed global picture of the interaction between host and pathogen may provide opportunities for identifying new targets for antimicrobial programs and vaccine development.

## Practical applications

We have reported the huge progress in diagnostics of microbial resistance and basic research in resistance and virulence achieved in recent years. Many of these advances have been transferred to protocols for practical diagnosis and will potentially lead to the development of new therapies against pathogenic bacteria. Such examples include the discovery of 34 unknown proteins of similar abundance found in both cell envelopes and membrane vesicles fractions in four *N. gonorrhoeae* strains (Zielke et al., 2014). Depletion of one of these, a homolog of an outer membrane protein LptD, was found to cause loss of viability in *N. gonorrhoeae*. These authors also identified another six predicted outer membrane proteins of unknown function. Loss of NGO1985, in particular, resulted in dramatically decreased viability of *N. gonorrhoeae* on treatment with detergents, polymyxin B and chloramphenicol, suggesting that this protein functions in maintaining the cell envelope barrier to permeability and may represent a new therapeutic target (Zielke et al., 2014).

Proteomic studies involving metabolism of virulent or resistant strains have been a source of potential therapeutic targets. For example, in a study investigating determinants of the biofilm condition of *A. baumannii*, Cabral et al. (2011) found that histidine metabolism (like Urocanase) was implicated in biofilm formation, as confirmed by gene disruption experiments. The authors proposed a model in which novel proteins are suggested for the first time as targets for preventing the formation of *A. baumannii* biofilms.

Vaccination has been revealed as an important strategy for reducing the incidence of infectious diseases. Immunoproteomics permits the characterization of putative vaccine candidates by combining proteomic analysis during infection together with serum identification of immunoreactive antigens. A recent review described examples of vaccine candidates that have been identified by immunoproteomics and have successfully protected animals against challenge when tested in immunization studies (Dennehy and McClean, 2012).

An exopolysaccharide and a protein-based biofilm expressed by two clinical strains of *S. aureus* have recently been studied by proteomic methods. Moreover, the number of bacterial cells inside a biofilm and in the surrounding tissue decreased after immunization with the biofilm matrix exoproteome in an *in vivo* model with a biofilm linked to infection (Gil et al., 2014).

## Concluding remarks

The method used for MS-based identification of proteins is constantly improving and has allowed progress from qualitative determination of proteins (i.e., presence or absence) toward quantitative studies of the proteome. However, the method still has many limitations. The inability to determine many proteins present at low concentrations, together with small number of proteins in the dataset may lead to errors in interpretation (e.g., associating complete biochemical pathways with virulence or resistance mechanisms). Comprehensive interpretation is also very difficult when the genome has not yet been obtained. This could be solved by using the highest resolution bottom-up approach and appropriate use of the knowledge base to improve the design of new studies and by the inclusion of specific statistical strategies to identify significant proteins. Next-generation sequencing should be linked with proteomic techniques. The combined use of different methods will cut down on the number of experiments required and improve the chances of identifying effective targets.

Basic aspects of bacterial growth and physiology are often ignored. Collaboration between a broad range of specialist including biochemists, biologists, and statisticians will be necessary, especially once metabolites can be synthesized. These metabolites must be assayed by metabolomic methods. Recent reviews of the relationship between metabolism and antibiotic resistance revealed global regulators that simultaneously alter virulence, metabolism and antibiotic resistance (Martínez and Rojo, 2011).

It can be difficult to coordinate and interpret the increasing volume of high resolution MS data obtained in the field of bacterial proteomics. Thus, an outstanding role for microbial bioinformatics is expected and more software packages for MS analysis must be shared. Attempts could also be made to merge several previous studies and select common patterns that ultimately enable better design of future experiments.

On the other hand, the multifactorial nature of bacterial infection must be investigated using several approaches, in a systems biology strategy. The coordinated use of different techniques including genomics, transcriptomics, and metabolomics, together with good standard proteomic methods will improve the capacity for detecting bacterial resistance, determination of resistance mechanisms and the understanding of virulence response in bacteria and host. This will hopefully help in the discovery of new biomarkers, vaccines, and drugs for combating bacterial infection worldwide.

In conclusion, in this review we summarize some of the applications of proteomic technology for diagnosing infections as well as performing basic studies for understanding antimicrobial resistance and virulence. This will enable discovery of new targets for novel and innovative antibiotics as well as the implementation of new therapies in the near future.

## Author contributions

FP, revised literature and wrote; GB, wrote and contributed with critical analysis.

## Funding

This work was funded by the European Community, FP7, ID:278232 (MagicBullet) and by the Plan Nacional de I+D+I 2008–2011 and Instituto de Salud Carlos III, Subdirección General de Redes y Centros de Investigación Cooperativa, Ministerio de Economía y Competitividad, Spanish Network for Research in Infectious Diseases (REIPI RD12/0015/0014) co-financed by the European Development Regional Fund “ A Way to Achieve Europe” ERDF. Additional funding was provided by the Fondo de Investigación Sanitaria (grants PI12/00552 and PI15/00860).

### Conflict of interest statement

The authors declare that the research was conducted in the absence of any commercial or financial relationships that could be construed as a potential conflict of interest.

## Abbreviations

ATCC: American Type Culture Collection
c-di-GMP: cyclic diguanylate
DIGE: difference gel electrophoresis
ICAT: isotope-coded affinity tags
iTRAQ: isobaric tags for relative and absolute quantification
LC–MS: liquid chromatography–mass spectrometry
MIC: minimal inhibitory concentration
MRSA: methicillin-resistant *Staphylococcus aureus*
MS: mass spectrometry
MSSA: methicillin-sensitive *Staphylococcus aureus*
Omp: outer membrane proteins
SDS-PAGE: sodium dodecyl sulfate polyacrylamide gel electrophoresis
SILAC: stable isotope labeling by amino acids in cell culture
SRM: selected reaction monitoring
TMT: tandem mass tags.
